# The evaluation of a physical health promotion intervention for people with severe mental illness receiving community based accommodational support: a mixed-method pilot study

**DOI:** 10.1186/s12888-021-03640-1

**Published:** 2022-01-04

**Authors:** Viola Kirschner, Natalie Lamp, Ümmügülsüm Dinc, Thomas Becker, Reinhold Kilian, Annabel Sandra Mueller-Stierlin

**Affiliations:** 1 Department of Psychiatry II, University of Ulm, Lindenallee 2, 89312 Günzburg, Germany; 2 Institute of Epidemiology and Medical Biometry, University of Ulm, Schwabstraße 13, 89075 Ulm, Germany

**Keywords:** Health promotion, Mental illness, Sheltered living, Motivational interviewing

## Abstract

**Background:**

Unhealthy lifestyle constitutes a cause of increased morbidity and mortality in people with severe mental illness. The aim of this mixed-method pilot study was to investigate the feasibility and preliminary effectiveness of an intervention to promote a health-conscious lifestyle in comparison to care as usual among people with severe mental illness receiving accommodational support in community settings.

**Methods:**

This was a prospective, quasi-experimental, controlled study over four six-month assessment points (t0, + 6 months, + 12 months, + 18 months) with 70 persons with severe mental illness receiving community based accommodational support. Mental health staff members of the housing facilities were trained in Motivational Interviewing and conducted a six-week health course with the intervention group participants in addition to care as usual. Next to the primary outcome - self-rated physical well-being (FEW 16) - anthropometric parameters and unhealthy behaviours (diet, physical activity, alcohol and tobacco consumption, and oral hygiene) were examined. Effectiveness analysis was conducted using mixed-effects regression models with propensity score adjustment to control for selection bias. One year after the end of the intervention, semi-standardized expert interviews were conducted with 12 of these employees and evaluated by content analysis.

**Results:**

The qualitative interviews with mental health staff underline the intervention’s feasibility in people with severe mental illness in sheltered housing, and the acceptability of and satisfaction with the intervention among mental health workers. But in this pilot study no superiority of the HELPS intervention compared to routine care could be demonstrated in terms of the investigated outcomes.

**Conclusions:**

The findings of this pilot study underscore the feasibility and acceptability of health promotion programmes based on Motivational Interviewing and highlight the need to further develop multi-modal programs according to the needs of the target group. Long-term and sustainable support for healthy lifestyles of people with severe mental illness receiving community mental health care requires multi-modal concepts and organisational change.

**Trial registration:**

DRKS00011659, date of registration was 2017/02/15; retrospectively registered as date of first enrolment was 2017/01/24.

**Supplementary Information:**

The online version contains supplementary material available at 10.1186/s12888-021-03640-1.

## Background

There is an urgent need for interventions addressing modifiable lifestyle risk factors to reduce the excess mortality among people with severe mental illness (SMI), as recently postulated by the Lancet Psychiatry Commission [1].

A growing number of studies has revealed an excess early mortality in people with SMI resulting in a 10 to 18 years shortened live expectancy [2–6]. This mortality gap, leading to a 2 to 3.5 times higher mortality rate in comparison to the general population [6, 7], has even widened over time [3, 5, 8]. Apart from suicide and accidental death, two-thirds of deaths in persons with SMI are due to physical illness [6] like cardiovascular disease [4, 9, 10], respiratory disease and several types of cancer [5, 7]. This high somatic co-morbidity and mortality can be attributed to environmental and organisational issues (e.g., inequalities in healthcare access and delivery, lack of resources, professional incompetence due to separation of mental health services from other medical services) [10–12] and to medication side effects (e.g. weight gain due to antipsychotics) [12–14]. Another important risk factor is an unhealthy lifestyle [15–17], partly related to the mental illness (e.g. lack of motivation and energy, need of support, isolation) [12]. Persons with schizophrenia tend to excessive dietary intake [18] and to have a diet poor in fibre and fruit and rich in saturated fat [19, 20]. These dietary patterns increase the risk of developing a metabolic syndrome [19] of which approximately one third of the people with SMI are suffering [21]. Moreover, these people are significantly more sedentary and less physically active compared to healthy controls [22]. There is an association between schizophrenia and tobacco smoking, greater frequencies of heavy smoking and high nicotine dependence with lower smoking cessation rates compared to the general population [23]. High mortality rates for chronic obstructive pulmonary disease, lung cancer, pneumonia and influenza implicate tobacco use as a major risk factor for premature mortality among persons with schizophrenia [7]. Individuals with mental illness are also more likely to consume alcohol [24], with doubled odds of risky alcohol consumption for patients with schizophrenic disorder and three times higher odds among patients with depression in comparison with the general population [15].

However, there are promising systematic reviews suggesting that health behaviour interventions have a positive impact on the physical health of people suffering from mental illness and providing evidence for the effectiveness of interventions based on physical activity and dietary behaviour for the treatment of cardiovascular risk factors in this population [25–27]. As an example, a systematic review and meta-analysis has revealed significant effects of nutrition interventions on weight loss, body mass index, waist circumference and blood glucose levels [28, 29]. For this reason, the Lancet Psychiatry Commission recommends the implementation of lifestyle interventions in routine care [1]. Given this background, the German S3 guideline “Psychosocial therapies for severe mental illness” also highly recommends multimodal health-promoting interventions with a focus on healthy eating and physical activity for people with SMI [30]. Nevertheless, this recommendation is only based on international evidence and no specific measures are mentioned. Therefore, the feasibility and effectiveness of lifestyle interventions for people with SMI needs to be evaluated in Germany so that they can be adapted and routinely implemented.

Already a decade ago, the European network for promoting the physical health of residents in psychiatric and social care facilities (HELPS) developed a physical health promotion toolkit which addresses nutrition, physical activity, smoking, alcohol consumption and oral health. The toolkit aims at enabling staff and people with SMI to identify their individual physical health risks and to develop strategies for lifestyle changes to manage a healthy lifestyle. For this purpose, the HELPS toolkit provides handouts and worksheets as well as self-assessment questionnaires and information about existing intervention measures, such as Motivational Interviewing (MI), which can be flexibly adapted to the patients’ needs [31]. Practitioners are not restricted to lifestyle experts as for example dieticians but can come from a wide range of clinical backgrounds. The aim was making the intervention easy and cost-efficient to implement in order to promote a widespread use [32, 33]..

Preliminary pilot data from the HELPS toolkit feedback survey support the feasibility and acceptability of MI for lifestyle changes in routine mental health care [32]. However, proof of effectiveness in a controlled study is still pending. As lack of fidelity in implementation is expected to be a key barrier to success, the HELPS network highlights the need for a thorough process evaluation [32].

The objectives of this study are to evaluate the feasibility and preliminary effectiveness of a lifestyle intervention based on the HELPS toolkit in a quasi-experimental, controlled pilot study in people with SMI receiving community based accommodational support and to identify factors that facilitate or impair the success of the intervention by means of semi-structured interviews with toolkit applicants.

## Methods

### Study design

A mixed-method research design was applied combining the collection of both quantitative and qualitative data. The quasi-experimental, controlled trial was conducted in Munich, Germany with 70 participants experiencing a mental disorder and living in facilities of community-based housing and day care. Preference-based group allocation to a lifestyle intervention based on the HELPS toolkit (intervention group) or to care as usual (control group) was performed. Data were collected at baseline and at three follow-ups after 6, 12 and 18 months.

As part of process evaluation, qualitative interviews were conducted with the trained MHWs. This mixed-method approach was selected in order to gain additional insights into the implementation, the barriers and enabling factors of the intervention by interviewing the mental health professionals apart from evaluating the preliminary effectiveness of the intervention by means of quantitative data.

The study was retrospectively registered on 2017/02/15 (DRKS00011659, first enrolment was on 2017/01/24) and was conducted as part of a doctoral thesis [34].

### Study population and sampling procedure

The committee “Health, Social and Care Planning” in Upper Bavaria has asked providers for community based accommodational support in Munich if they would be interested in piloting a health-promoting intervention. Finally, recruitment of participants took place at eight facilities by staff on site who screened clients for eligibility (e. g. linguistic and intellectual abilities, alcohol addiction) based on the client record.. All clients fulfilling the eligibility criteria were informed about the study by staff of these facilities and a meeting for the baseline assessment was scheduled with all clients willing to participate. At the beginning of this meeting, research workers provided the clients with verbal and written information regarding the study and asked the clients to give written informed consent.

Inclusion criteria were age between 18 and 65 years, presence of mental illness, in assisted living and in need of improving health-related lifestyle behaviour according to MHWs’ judgement. Owing to the project funding, only persons with a membership in a specific health insurance company (AOK Bayern) were included. With 4.5 million members (equivalent to one third of Bavarian residents), AOK Bayern is the largest statutory health insurance company in Bavaria [35]. Patients with insufficient linguistic and intellectual abilities were excluded and patients with alcohol addiction were not allowed to participate in the study.

### Care as usual

In Germany, care as usual (CAU) includes medical in- and outpatient hospital treatment, ambulant treatment by office based family doctors and specialized physicians including psychiatrists, ambulant psychotherapy, other ambulant therapies and medication all financed by the mandatory health insurance. In addition, accommodational and several other types of community based support for people with severe mental illness is financed on the basis of taxes by the communities or other regional authorities. The participating facilities offer several types of ambulant accommodational support or sheltered housing. The staff members offer support with living and self-care, as well as social issues (e.g. social relations, leisure activities) and medical care (e.g. attendance at appointments with doctors, talks in personal crises). In sheltered housing facilities there is support with shopping and cooking and various physical activities. Nevertheless, it is assumed that lifestyle behaviour and somatic needs are insufficiently addressed in current psychiatric care [10, 36].

### Intervention

The intervention aimed at promoting a health-conscious lifestyle of people with severe mental disorders.

The lifestyle intervention was delivered by mental health workers (MHWs) who were interested in implementing health promotion measures. In total, 17 MHWs, mainly social workers, at eight facilities received a two-day training to use the HELPS toolkit and in particular on how to practice MI. The course was led by the scientist who coordinated the development of the HELPS toolkit (a psychotherapist with specific training in MI) and the scientist coordinating the pilot study (a nutritionist with specific training in MI). Feedback evaluation revealed overall satisfaction with the organization of the training, the comprehensibility and the trainer’s performance. However, it was occasionally stated that the amount of time, the applicability in practice and the benefit for further professional life were only partially satisfactory.

The MI module of the HELPS toolkit [31] enables staff and people with SMI to develop strategies for lifestyle changes according to the trans-theoretical model of behaviour change (pre-contemplation, contemplation, preparation, action, maintenance and relapse) developed by Prochaska and DiClemente [37, 38]. MI is a collaborative communication style, based on four central overlapping processes (engaging, focusing, evoking and planning), that is designed to support intrinsic motivation for and commitment to a specific goal leading to sustained behaviour change [39].

The MHWs’ task was to create supporting alliance that will enhance clients’ motivation to change and to support them in setting individual targets in one of five domains (nutrition, physical activity, smoking, alcohol consumption or oral health) and in developing strategies for improving health behaviour [32]. They offered a 6-week health promotion programme to the participants of the intervention group. The health promotion programme was provided as face-to-face meetings at recruiting facilities in individual or in group setting, mainly on a weekly basis with each session lasting 1 to 2 h. In addition, participants of the intervention group, facilitators and MHWs were encouraged to address the goals set and health behaviour between the programme appointments and subsequent to the programme in the context of care as usual in sheltered housing. Intervention group participants could use all other services available as part of care as usual.

### Outcomes and assessments

Data were collected at baseline and three follow-ups at 6 months, 12 months and 18 months after baseline (Table 1). The time windows for the follow-up examinations ranged from plus / minus 4 weeks to the scheduled time.

**Table 1:**
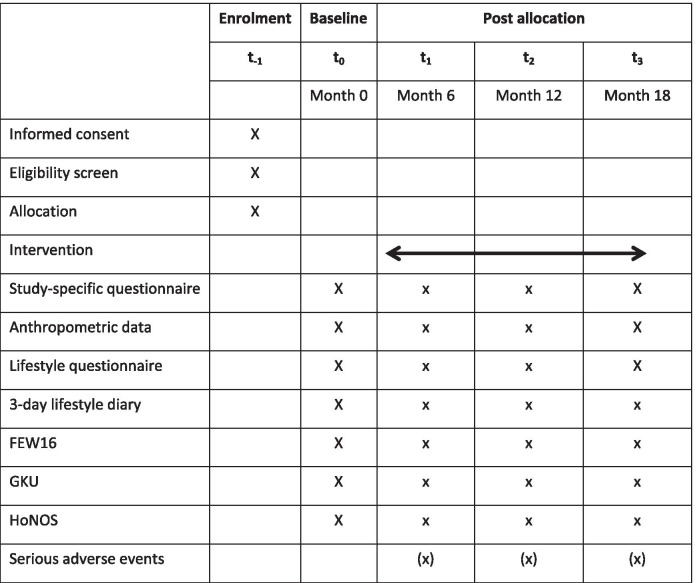
Patient Enrolment, Intervention, and Assessments Schedule

The data were collected in personal interviews through self-assessment questionnaires and research worker rating scales. Furthermore, anthropometric parameters were determined and three-day food and activity records were conducted. The interviews took place in the mental health care facilities or at the participants’ homes by research workers of Ulm University.

Due to the preference-based allocation, blinding of the participants was not feasible. The research workers responsible for data collection were not informed about allocation group, but it could not be ruled out that the participants revealed the allocation group during the assessments.

At baseline, sociodemographic data as well as the medical history, including somatic illnesses and medication, were collected.

The primary outcome was change in physical well-being (FEW16) [40] over 18 months. The FEW16 questionnaire measures habitual physical well-being, which is not defined merely as the absence of illness, pain or functional impairment. The FEW16 questionnaire is a self-assessment tool with 16 items covering the four dimensions “stress resistance”, “ability to enjoy”, “vitality”, and “inner peace”. Each item is assessed on a scale from 0 to 5 and the mean value for the overall questionnaire and for the individual dimensions is calculated. A high score indicates a high level of physical well-being [40]. Physical well-being as a rather generic outcome was chosen because it is assumed that changes in any of the five lifestyle domains (nutrition, physical activity, tobacco and alcohol consumption and oral hygiene) would have a positive effect on this outcome.

In contrast, secondary outcomes were rather related to specific lifestyle domains and mental health. Body mass index (BMI) and waist-to-hip ratio were monitored in all participants. In addition, the participants were asked about health-relevant features of their lifestyle (nutrition, physical activity, tobacco and alcohol consumption and oral hygiene) using a questionnaire that is based on large epidemiological studies on health behaviour (NVSII, DEGS1) [41–43].

Participants were asked to record each individual food and beverage consumed over 3 days in a guided diary, that lists the weight/volume of standard serving sizes for a wide range of food items and beverages [44]. Nutritional value analyses were carried out using DGExpert software. The Healthy Dietary Indicator (HDI) was determined as per seven nutrient-based and two food-based items [45]. The HDI is 0 to 9 points, with 9 points indicating optimal dietary behaviour.

Moreover, a Food Frequency Questionnaire covering 32 food groups was used to assess dietary patterns. The food frequency of each food group was rated as poor (0), adequate (1), or optimal (2). A high sum score, which is also referred to as the Dietary Quality Index, indicates a good dietary quality [46].

In addition, participants were asked to record activity levels on an hourly basis over a three-day period. The mean duration of physical activity per day was derived based on this diary. A Sport Quality Index was assessed in a similar way as the Dietary Quality Index based on an Activity Frequency Questionnaire covering eight activity levels. A high Sport Quality Index indicates good activity levels.

The Health of the Nation Outcome Scale (HoNOS) was used to measure the clinical and psychosocial impairment of the client, independent of diagnosis [47–49].

Data capturing and data management was performed centrally at Ulm University using IBM SPSS 25. Questions that arose during data entry were resolved with the research workers and, if required, with the study participants.

### Sample size calculation

The sample size calculation was performed for the change in the physical well-being total score over 18 months. The sample size estimation was based on the purpose to detect a moderate effect size (f = 0.5) at *p* ≤ 0.05 with a power of 0.90 using an univariate analysis of variance (ANOVA) for four repeated measurements and assuming a dropout rate of 15%.

### Statistical analysis

Data analysis was carried out using SAS 9.4 and STATA 14.

Due to the organisational conditions of the study, a randomised allocation of the study participants was not possible and preference-based group allocation occurred through the participants themselves. Propensity score adjustment was used to control selection bias [50]. Propensity scores were estimated based on a logistic regression model including all variables for which group differences (*p*-value < 0.05) were shown in baseline assessment. Potential independent variables for propensity score estimation include the clients’ medical history and clinical status as well as sociodemographic and lifestyle characteristics [51].

Linear mixed-effect regression models for panel data [52] with a random time effect, a fixed group effect and an interaction effect between time and study group adjusted for propensity scores [53] were computed for primary and secondary outcome variables. The group by time interaction reflects the intervention effect. The repeated measurements for patients clustered according study centres were taken into account in the covariance structure. The analysis was conducted on the intention-to-treat population. Missing values were taken into account by weighting of the parameter estimation [54, 55].

### Qualitative interviews

In order to gain a comprehensive insight into the implementation of the intervention, semi-structured interviews were conducted with the trained MHWs who led the health promotion courses. The interviews were conducted between the third and fourth assessment of the quasi-experimental controlled study, i. e. about 1 year after the end of the health promotion course.

A semi-structured interview guide was developed around 1) the implementation of the intervention, 2) positive and negative experiences with the intervention and 3) appraisal with a view to the future application of the intervention.

The interviews were conducted by telephone, audio-recorded electronically and transcribed verbatim in German. All transcripts were checked for accuracy and summaries were produced by VK.

### Qualitative analysis

Using the software MAXQDA 11, structuring qualitative content analysis based on Mayring [56] was applied. Firstly, a summary was written for every interview in order to get a good overview about the data. Afterwards, main categories were derived deductively from the topic guide. In the next step, sub-categories were developed by inductive coding procedures. This meant that every text section was coded by summarising its content in one or more codes. Afterwards, these codes were structured by developing sub-categories. During the constant analysis of new data, these sub-categories were continuously revised and adapted. The analysis was documented in a research diary by the first author (VK) and presented and discussed in a weekly meeting (VK, AMS) to ensure consensual coding.

## Results

### Study population and study flow

**Fig. 1:**
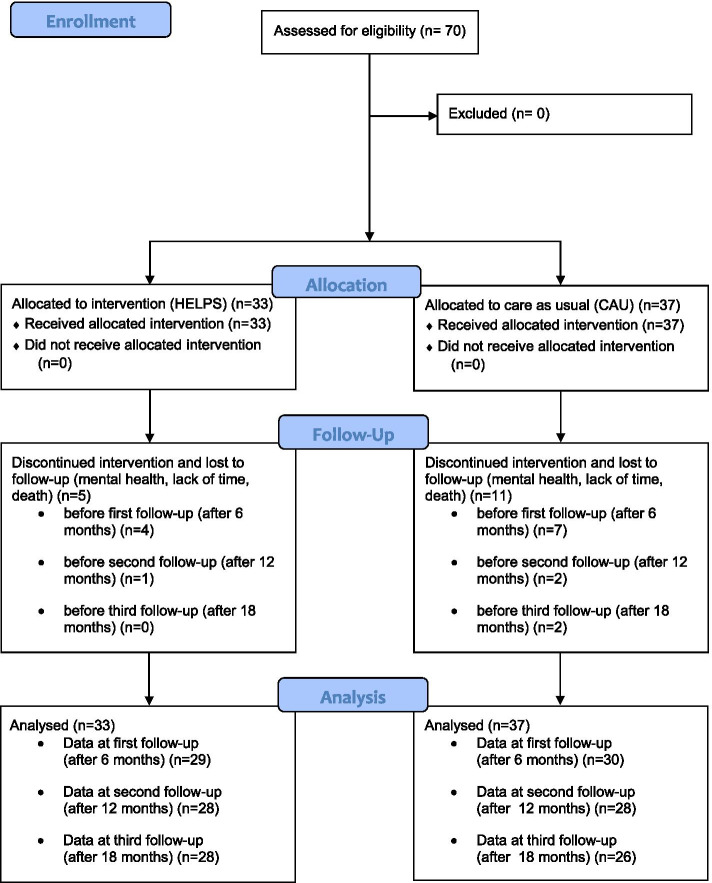
Study flow

Recruitment of 70 patients (intervention = 33, control = 37) took place between January 2017 and June 2017 (see Fig. 1 and Table S1, S2 and S3). Study closing date was in December 2018. The dropout rate amounts to 22.9% (*n* = 16), while the highest drop-out rate was observed between baseline and the first follow-up assessment (*n* = 11; 15.7%).

On average, the participants were 50 (sd = 11.8) years old at baseline assessment and most of them were men (61.4%). There were no significant differences in age and gender between the two groups. Only one participant (1.4%) in the intervention group stated that he or she lived in a stable partnership. Participants in the intervention group had significantly more contact with their families (p (CAU) = 48.6%, p (HELPS) = 75.8%, *p* = 0.020) and they also reported having children more often, although not significantly (p (CAU) = 18.9%, p (HELPS) = 39.4%, *p* = 0.058).

The average age at the onset of the disease (main diagnosis) was 31.5 (sd = 13.82) years, which results in an average duration of the disease of about 18.8 (sd = 13.1) years. Half of the participants in the intervention group (50.0%) reported more than one mental illness. The most frequent diagnoses refer to the group of schizophrenic disorders (F20-F29 according to ICD-10; 57.1%), affective disorders (F30-F39 according to ICD-10; 37.1%) and neurotic, stress or somatoform disorders (F40-F48 according to ICD-10, 22.9%). The participants had an average of seven to eight previous inpatient stays in a mental health clinic (Median = 5) and 21% of the participants had an inpatient stay in the 12 months prior recruitment. The majority of participants (91.4%) reported having taken medication due to the mental illness in the 18 months prior to baseline assessment.

No significant baseline differences were found between study groups regarding physical health outcomes (Table 2 and Table S4). The participants reported a moderate level of physical well-being (mean (CAU) = 2.82 (sd = 1.20), mean (HELPS) = 2.77; sd = 1.26)). More than two thirds of the study participants suffered from one or more physical diseases at baseline assessment. With an average BMI of 32.63 (sd = 8.27) kg/m^2^, the majority (82.6%) of the study participants were classified as overweight and more than half (56.5%) as obese according to the definition of the World Health Organisation (WHO). Almost all participants (92.5%) had a waist circumference which, according to the WHO, is associated with an increased risk of metabolic complications. Moreover, the baseline values indicate that the intervention group (mean = 35.39, sd = 8.39) participants were initially more convinced that their behaviour could influence their physical health compared to participants of the control group (mean = 39.89, sd = 7.87) (*p* = 0.024).

**Table 2:** Results of mixed-effects regression models for the primary outcome

Physical well-being	B	Se	*p*	95% - CI
intercept	2.4354	0.3271	**<.0001**	1.7826–3.0883
group	−0.4436	0.3408	0.1959	−1.1192 - 0.2320
time	−0.0799	0.0586	0.1784	−0.1972 - 0.0375
time * group	0.1365	0.0825	0.1011	−0.0271 - 0.3001
PS	1.3194	0.6116	**0.0332**	0.1069–2.5319

group = mean difference between control and intervention group at baseline

time = linear change from t0 to t3 in control group

group x time = difference in linear change between control and intervention group

PS = estimated coefficient for propensity-score adjustment

As only few data were missing, no multiple imputation of missing data was performed. However, independent variables were single imputed to estimate a propensity score for each participant. These imputed values were not used for further analysis. The contact frequency with the family and the health-related locus of control were used for propensity score estimation, because baseline differences between groups were found for these variables at a significance level of 5%. The common support area of the propensity score ranges from 0.055 to 0.685 and does not comprise 20% of study participants (n (CAU) = 0, n (HELPS) = 12).

The core component of the intervention was an health promotion course, which took place over 6 weeks in individual (28.6%) or group settings (71.4%). The majority of intervention group participants (82.1%) attended all six sessions. However, complementary support by means of short individual contacts during and after the health promotion course was only offered in single cases, mainly for reasons of limited time resources.

### Outcome effects

The results of the mixed regression for physical well-being (Table 2) show neither a significant interaction effect between time and treatment group membership (b = 0.14; *p* = 0.1011), nor main effects of time (b = − 0.08, *p* = 0.1784) or treatment group (b = ¬0.44, *p* = 0.1959).

Also with regard to the remaining outcomes, no significant main or interaction effects were found in terms of time and group affiliation (see Supplementary Table S4). The secondary outcomes include psychosocial impairment as well as body mass index, waist-to-hip ratio, dietary behaviour and physical activity.

### Qualitative results

From June to July 2018, twelve (70.6%) of the 17 trained MHWs took part at qualitative interviews. At least one trained MHW from each facility was interviewed. The main reasons for non-attendance were job changes and parental leave. The average duration of the interviews was 30 min, with a minimum duration of 23 min and a maximum duration of 38 min.

The key themes derived from the analysis included (1) implementation of the intervention, (2) enabling factors, (3) barriers, (4) observed changes in service users’ attitudes and behaviour and (5) future of the intervention. The results are presented under these key themes and illustrated by quotations taken from the interviews to provide detailed insights.

Themes and subthemes are summarised in Table 3.

**Table 3:** Themes and subthemes derived from qualitative content analysis

Themes	Subthemes
Implementation of the intervention	Phases of behaviour change
	Implementation of the six-week health promotion course
	Implementation of the aftercare
Enabling factors	Regarding the implementation
	Regarding the patients
	Regarding the facility
Barriers	Regarding the implementation
	Regarding the facility
	Regarding patients’ characteristics
Observed changes in attitude or behaviour	Observed changes related to health behaviour
	Observed changes beyond health behaviour
	Negative observed changes
Future of the intervention	Positive evaluation
	Integration into daily work

#### Implementation of the intervention

##### Phases of behaviour change

The MHWs reported that a majority of the patients went through all phases of the trans-theoretical model of behaviour change at their own pace, with the preparation phase and the relapse being the most critical phases.

As concerns the preparation phase, the MHWs reported that patients initially focused rather on diet or physical activity than on smoking, alcohol consumption or oral health. The overriding goals could usually be named very quickly. However, MHWs reported of patients’ challenges in *“specification of goals”* (P09) and thus reducing their own expectations to small goals within the chosen thematic area. The MHWs reported that it was difficult for the patients to choose realistic goals that were not set too high. The MHWs reported that the patients finally worked out *“very, very low”* (P01) and *“small goals that are really achievable” (P12)*. For example, the patients tried to reduce the consumption of beverages containing sugar or caffeine and to increase the consumption of healthy and fresh foods like salad. The goals for increasing physical activity were very specific, for example a *“daily walk”* (P10) or *“cycling once a week on Wednesday” (P10)*.

The majority of the MHWs reported relapses, often occurring repeatedly with a high need for support from the MHWs. According to the statement of one MHW, relapses occurred *“parallel to […] [the] mental and physical health condition”* (P05) of her patient. Especially regarding diet, relapses were experienced as a big problem and some MHWs assumed that this is due to the emotional significance of eating.

##### Implementation of the six-week health promotion course

As reported by the trained MHWs, the health promotion programme was mostly carried out in a group setting with two or three patients. The MHWs experienced positive group dynamics, interaction and dialogue as the main advantages of this setting. In this context, the MHWs reported about the sympathy among the patients and the valuable advices they could exchange for dealing with the common problems on the way to improve their lifestyles. Contrary to the apprehensions of some MHWs, the patients had no problems opening up in an unfamiliar group.

*“Everyone knew the problem and then they reassured each other a little bit.”* (P06)

*“I found it incredibly valuable when the supervisors hold themselves back a little and they [the patients] got tips from the other patients, I had the impression that the patients could accept this more easily and also implement it more easily than if we as professionals gave them instructions. That it will be easier for them to accept that.”* (P06)

The disadvantages observed in the group setting resulted mostly from patients’ individual needs and cognitive difficulties impairing their participation in group discussions. MHWs also reported difficulties in organising the appointments of the group sessions and ensuring the participation of all patients. Some MHWs who decided to conduct the health course in an individual setting noted that organisation was simplified by integrating it into meetings as part of care as usual. Two MHWs felt that this setting, although being more intensive and stressful for the patients, could provide better care and individual support.

##### Implementation of the aftercare

Only three MHWs reported that they had addressed the predefined behavioural goals between the programme appointments. In their consideration, these additional meetings helped to tailor the issues discussed in the group sessions to the patients’ individual needs.

The intensity of these aftercare meetings varied greatly. If the MHWs providing the health promotion course was also providing the usual mental health care, regular mental health care meetings were used to discuss lifestyle issues. In contrast, if the MHW was not involved with the regular mental health care, aftercare sessions took place only sporadically due to limited time resources. In these cases MHWs reported that the patients *“felt left alone”* (P06) with relapses *“in times of crisis”* (P06).

#### Enabling factors

Some factors promoting the implementation of the intervention already emerge from the prior remarks, like for example the advantages of the setting used.

In general, the support of their facility and colleagues was experienced as beneficial by MHWs as there is need for time and personnel resources to provide the intervention.

The MHWs also felt that a good relationship and familiarity with the patients as well as the support of the MHWs responsible for regular care had positive effects on the patients’ behaviour change process.

Additionally, the MHWs identified the patients’ willingness and motivation to participate in the intervention as an important factor promoting behaviour change:

*“So what functioned well, one noticed, was that the residents were present and that they wanted something changed. And their diet really has changed... One could see the success.”* (P12)

#### Barriers

As main barriers against the successful implementation of the programme interviewees mentioned the lack of a constant aftercare and disadvantages of the health promotion programme setting, like for example the insufficient support of individual needs during group sessions.

At the organisational level, communication problems were reported regarding the distribution of information material on the intervention and exchange of information between staff about individual goals and action plans. The MHWs also mentioned time problems that arose in the organisation and implementation of the intervention in addition to the regular workload.

When explicitly asked for barriers, the MHWs emphasized psychological stress caused by the patients’ *“basic problem”* (P02) and their general living situation as main factors impeding lifestyle changes. Lack of drive, side effects of drugs and mental impairment such as “*states of tension and fear”* (P09) prevented the patients from pursuing their goals and provoked relapse or even dropout. Furthermore, cognitive impairments as a result of illness or drug side effects were often mentioned as barriers. These *“very, very chronically ill”* (P01) patients had difficulties in making use of the toolkit and following group discussions due to a *“lack of both concentration and perseverance”*.(P02).

In addition, some patients were described as very uncommunicative which was regarded as complicating the identification of needs and goals for lifestyle change. In some cases MHWs considered their patients’ reluctance to open up as resulting from being ashamed about their behavioural problems. Patients’ inability of dealing with perceived failure was regarded by some MHWs as very challenging for some service users and as reason for the programme dropout.

Further barriers considered by MHWs were old habits that impeded patients from trying out something new, especially with regard to diet and a lack of reliability during the process of behaviour change.

#### Observed changes in attitude or behaviour

##### Observed changes related to health behaviour

The majority of interviewed MHWs observed positive changes of their patients’ attitudes and behaviours. First, MHWs reported that the patients’ willingness to deal with their lifestyle was raised, even among patients who were initially described as *“very, very closed”* (P12) and with *“reservations”* (P12). The MHWs emphasized that the patients became aware of their unhealthy habits, most of them for the first time in their life. Frequently mentioned during the interviews was the subsequent growing health-consciousness with the focus on diet and physical activity:

"*Well, before that, one of them only drank sugary drinks and no water at all, and he didn't even see that this was a problem. [...] In any case, he now has an awareness of where he is crossing the line of what is healthy. [...] They now have more awareness of what would be healthy, they sometimes implement some of it, but of course they don't always manage to do it.”* (P10)

The MHWs provided a lot of examples of how in their view the patients’ raised awareness led to changed health behaviour. Regarding diet and physical activity, the MHWs described a wide range of observed behaviour changes, ranging from very small, individual steps to relevant, measurable effects:

*"One of them lost ten kilos and now cooks fresh food in the evening, eats mostly vegetables and fruit and no sweets anymore.”* (P10)

However, such measurable effects, like weight reduction or lowering of blood sugar levels, were only rarely reported. In most cases the behavioural changes were perceived as having taken place *“in really small steps”* (P08), so that the patients are now *“one mini-step further”* (P02):

*“One of the residents who took part in the project no longer drinks three litres of cola mix. Now he only drinks one and a half litres.”* (P10)

*„For example, one patient started taking the stairs instead of using the lift, walking through the city at least once a day, or getting off the bus one stop earlier.“* (P06)

With regard to oral hygiene, MHWs reported that regular tooth brushing was a common and often accomplished goal:

*“The patients reported that they made significant progress within the six weeks. There were patients who did not brush their teeth at all, patients who brushed only once a week, and patients who brushed only once every 14 days. It was then possible for these patients to brush their teeth in the evenings.”* (P06)

##### Observed changes beyond health behaviour

In addition, MHWs reported having observed various changes in patients beyond health behaviour. MHWs had the impression that the intervention promoted the patients’ independence and personal responsibility. The problem-solving strategies and approaches developed during the health promotion course strengthened the participants’ self-confidence and self-efficacy, as observed by the MHWs:

*“But the way of thinking about it [behaviour] and solving the problems associated with it, I think, has changed.”* (P08)

MHWs also described an increased self-esteem when the patients achieved their personal health goals. Besides, the MHWs perceived the patient-centred approach of the intervention and MI as very helpful in order to improve the patients’ willingness to talk about difficult personal issues even beyond their lifestyle.

Moreover, the MHWs supposed an impact on the patients’ social life as they observed an exchange between the patients even after the health promotion programme and small but positive changes in their daily structure. One MHW observed an increased drive of her patients especially shortly before the health promotion course sessions, because the patient wanted to report her success to the group.

Furthermore, MHWs pointed out that the patients’ awareness of the link between physical and mental health was raised and that the patients realised that “*if you feel good in one direction, [...] it works out for the other side too”.* (P04).

##### Negative observed changes

Only a few negative intervention effects were observed by the MHWs. Some patients were unhappy with the intervention as they missed to receive clear behavioural guidelines and didn’t want to identify their personal goals and action plans. Other patients didn’t like the confrontation with their own misbehaviour and felt stressed due to not fulfilled expectations and goals they had.

#### Future of the intervention

In general, the intervention was positively evaluated by the MHWs. They considered the toolkit and Motivational Interviewing as very helpful and the focus of the project on a healthy lifestyle as very important as *“the physical health of mentally ill people [...] is very impaired”* (P05) and their health behaviour is *“deficient”* (P05). Due to their positive experiences, many MHWs stated that they would integrate both the Motivational Interviewing and the topic of a healthy lifestyle into their daily work.

## Discussion

The HELPS project aims at the promotion of physical health among people with SMI based on MI. This pilot study was the first to evaluate its feasibility and preliminary effectiveness over 18 months in German mental health and social care facilities.

In the mixed-effects regression models no significant effects on primary (physical well-being) and secondary outcomes (BMI, physical activity and diet) were shown. Even though this study could not demonstrate the effectiveness of the intervention, important knowledge was gained about feasibility and in particular facilitators and barriers in the implementation of lifestyle interventions for people with SMI.

The HELPS intervention is based on the technique of MI [32] that seeks to strengthen the patients’ intrinsic motivation for behaviour change focusing on empowerment and autonomy and is flexibly adaptable to individual and contextual needs [32]. This approach is well-grounded in theory and research. A systematic review and meta-analysis evaluated the efficacy of MI across medical care settings and found a statistically significant and positive impact on outcome measures like quality of life, body weight, sedentary behaviour, alcohol consumption, smoking abstinence and dental caries [57]. No statistically significant effect could be found on healthy eating [57]. Other reviews also confirm positive effects of MI in respect to health-related outcomes and behaviour changes [58–62]. Nevertheless, it must be admitted that studies among people with SMI evaluating the effectiveness of MI to improve health behaviour are rare. However, MI has shown great promise in this target group for other outcomes like addiction treatment [63] and help seeking for mental health issues [64].

In general, the findings of a systematic review including 42 articles indicate that health behaviour interventions can result in significant improvements in health behaviour and health outcomes among people with mental illness by reducing poor dietary habits, physical inactivity, smoking and alcohol abuse [18]. Another review including 108 studies found beneficial effects of behavioural and pharmacologic interventions on obesity among persons with SMI [38]. However, one explanation for the lack of proof of effectiveness might be that MI alone is not enough to achieve the desired changes, especially in this target group who has an increased need for support in everyday live and with a predominantly low level of education. Other programmes are mostly based on multi-modal concepts including MI only as one component, like for example the “In SHAPE” individualised health promotion programme for people with SMI [65, 66]. This programme combined MI with a fitness club membership, exercise instructions and nutritional education [65] and achieved clinically significant weight loss (≥5%) or increased fitness in approximately half of the participants [66, 67]. Already during the HELPS toolkit development, patients and staff emphasized in focus groups the importance of education and information on a healthy lifestyle like diet and physical activity for health promotion [32]. A recent systematic review and meta-analysis of 20 studies also identified educational meetings and health information systems as effective intervention strategies for preventive care provision for chronic disease risk behaviours in mental health settings [68].

Another effective health promotion programme for people with severe mental illness is the Australian “Keeping the Body in Mind” (KBM) programme, a multidisciplinary lifestyle intervention that addresses dietary behaviour and physical activity of individuals with first-episode psychosis [69]. The KBM programme consists of health coaching, nutrition counselling and supervised exercise. The individualised approach in the KBM programme combines MI with the teaching of practical skills like meal planning, purchasing ingredients and cooking [70]. The programme’s feasibility and effectiveness were shown in pilot studies [71, 72] and so, for example, it was shown that the KBM programme is effective to attenuate antipsychotic-induced weight gain in first-episode psychosis [69]. The KBM programme and the HELPS intervention differ, among others, in two aspects: First, while the KBM programme is provided by qualified physical health professionals (dietitians, exercise physiologists etc.), the HELPS intervention is delivered by trained MHWs. The KBM approach to embed lifestyle experts within mental health teams [73, 74] is supported by results of a systematic review that found that dietary interventions in SMI are most effective when delivered by a dietitian [29].

Second, the target population of both programmes differ markedly. While the KBM programme takes place at the start of antipsychotic pharmacotherapy, the HELPS intervention was implemented in facilities for accommodational support, where people are predominantly chronically ill. The importance of an early integration of lifestyle interventions into the treatment of severe mental illness was highlighted by Teasdale et al. who found in a systematic review the largest effects of dietary interventions at the start of antipsychotic pharmacotherapy [29]. For this study, it also implicates that it’s challenging to achieve measurable effects and prove the effectiveness of the intervention among our study population with an average duration of illness of 19 years.

Further factors impeding the improvement of the chosen outcomes in this study should be considered: It can be assumed that the intensity and duration of the intervention weren’t sufficient and that MI wasn’t implemented consequently taking into account the interviews with toolkit applicants. The two-day training in MI for toolkit applicants was probably too short as well as the six-weeks health course for participants of the intervention group, as reviews indicate an average intervention length of 20 weeks [28] to 27.4 weeks [25] in comparable health behaviour interventions.

However, positive effects on health weren’t directly visible or even measurable. According to the interviewees, problem and health awareness in general was raised, which in turn enhanced motivation and initiated first positive changes in behaviour. In addition, the MHWs observed a promotion of independence and self-responsibility and an improvement of the patients’ social life. However, these assumptions cannot be verified within the current study; these process outcomes should definitely be investigated in further studies on MI-based physical health promotion programmes.

Toolkits applicants concluded that they will continue to promote a health-conscious lifestyle of their patients by MI following the HELPS toolkit. But, as physical health promotion isn’t the focus of these facilities providing accommodational support, MHWs mentioned time problems related to the organisation and implementation of such an intervention in addition to the regular workload. This is in line with the requirement that health promotion must permeate the entire organisation of mental health care [75]. Consequently, it must be discussed how policies might support the promotion of physical health in routine care.

### Strengths and limitations

This is one of the rare pilot studies of health promotion interventions in people with severe mental illness in Germany. The major strength is the mixed-method design that allows for deep insight in implementation factors, mainly based on the qualitative part. Although this controlled, quasi-experimental pilot study failed to demonstrate superiority of the intervention over care as usual, the toolkit applicants reported in the interviews mostly positive changes in the lifestyle of their clients, so several small individual success stories.

But four limitations should be considered:

- First, this pilot study is not powered to reveal small intervention effects, which might be assumed as a result of poor implementation. Moreover, heterogeneity of the sample in terms of medical history, current health care (especially medication), and other sociodemographic issues might impede the proof of effectiveness and further studies with larger samples are required.
- Second, all outcomes except the physical well-being were targeting only specific lifestyle domains. During intervention, the participants focused on different domains (diet, physical activity, smoking, alcohol consumption or oral hygiene) according to their individual health risk and consequently, intervention effects can only be expected in this individual domain. However, the small sample size doesn’t allow subgroup analysis.
- Third, a selection bias due to the lack of randomisation can’t be definitely ruled out. Less than two thirds of the intervention group participants (20 out of 33) are in the common support area. This questions the effectiveness of the propensity score adjustment and indicates that, despite the statistical control, a distortion of the results can be expected.
- Fourth, the facilities providing community based accommodational support (and the intervention) for the trial participants were no random sample of all local service providers. Therefore, it is likely that the participating facilities have a higher interest in health promotion activities than the average and thus, findings might not be generalizable to other settings with less engaged staff.

## Conclusions

Despite the recommendation to promote the physical health of people with severe mental illness in relevant treatment guidelines [30], to date such interventions have hardly been neither applied nor explored in Germany. This is the first pilot study to investigate the feasibility and acceptability of a simple lifestyle intervention in sheltered living for people with severe mental illness. The pilot study indicates the feasibility of this MI-based intervention, even though its effectiveness has not been shown. The results of additional qualitative interviews with implementing MHWs reveal the need for further development of the MI-based intervention into a multi-modal program, which also aims at organisational change.

Subsequently, a larger trial is warranted to evaluate effectiveness of the intervention on different lifestyle domains.

## Supplementary Information

**Additional file 1: Table S1.** Sociodemographic characteristics at baseline. **Table S2.** Clinical characteristics related to mental health at baseline. **Table S3.** Clinical characteristics related to physical health at baseline. **Table S4.** Results of mixed-effects regression models for secondary outcomes.

## Data Availability

The datasets generated and/or analysed during the current study are not publicly available due to the used data protection declaration, but are available from the corresponding author on reasonable request.

## Abbreviations

AOK: Statutory health insurance institution (Allgemeine Ortskrankenkasse)
AWO: Arbeiterwohlfahrt
BMI: Body mass index
CAU: Care as usual
DEGS: Studie zur Gesundheit Erwachsener in Deutschland
FEW16: Physical well-being
HELPS: European network for promoting the physical health of residents in psychiatric and social care facilities
HoNOS: Health of the Nation Outcome Scales
ICD-10: International Classification of Diseases (Version 10)
KBM: Keeping the Body in Mind
KBO: District hospitals in Upper Bavaria
MHW: Mental health worker
MI: Motivational Interviewing
NVSII: Nationale Verzehrsstudie II
SMI: Severe mental illness
WHO: World Health Organisation
