# Supplementary material for: The evaluation of a physical health promotion intervention for people with severe mental illness receiving community based accommodational support: a mixed-method pilot study
Source: BMC Psychiatry. 2022 Jan 4;22:6. doi: 10.1186/s12888-021-03640-1 (PMC8728992; doi:10.1186/s12888-021-03640-1)
Supplement: Supplementary file 1 — Additional file 1: Table S1. Sociodemographic characteristics at baseline. Table S2. Clinical characteristics related to mental health at baseline. Table S3. Clinical characteristics related to physical health at baseline. Table S4. Results of mixed-effects regression models for secondary outcomes. [file 12888_2021_3640_MOESM1_ESM.docx]

**Table S1: sociodemographic characteristics at baseline**

|  | **total** | | **CAU** | | **HELPS** | | **p** |
| --- | --- | --- | --- | --- | --- | --- | --- |
| age (years); m (sd) | 50.34 | (11.83) | 50.43 | (11.73) | 50.24 | (12.11) | 0.947 |
| children; n (%) | 20 | (28.6%) | 7 | (18.9%) | 13 | (39.4%) | 0.058 |
| female; n (%) | 27 | (38.6%) | 15 | (40.5%) | 12 | (36.4%) | 0.720 |
| education, more than 9 years of education (junior high school or higher education); n (%) | 23 | (32.9%) | 12 | (32.4%) | 11 | (33.3) | 0.936 |
| vocational training; n (%) | 43 | (63.2%) | 20 | (55.6%) | 23 | (71.9%) | 0.164 |
| living in a stable partnership; n (%) | 1 | (1.4%) | 0 | (0.0%) | 1 | (3.0%) | 0.286 |
| contact with family more than once a month; n (%) | 43 | (61.4%) | 18 | (48.6%) | 25 | (75.8%) | **0.020** |
| contact with friends more than once a month; n (%) | 58 | (82.9%) | 28 | (75.7%) | 30 | (90.9%) | 0.091 |

n = absolute frequency; % = relative frequency; m = mean; sd = standard deviation of the mean / Pearson Chi^2^ test for categorical variables and t-test for continuous variables; significant effects (p <0.05) are printed in bold

**Table S2: clinical characteristics related to mental health at baseline**

|  | **total** | | **CAU** | | **HELPS** | | **P** |
| --- | --- | --- | --- | --- | --- | --- | --- |
| duration of disease (main diagnosis) (years); m (sd) | 18.84 | (13.07) | 19.11 | (13.14) | 18.55 | (13.18) | 0.859 |
| age at onset of disease (main diagnosis); m (sd) | 31.50 | (13.82) | 31.32 | (12.76) | 31.70 | (15.11) | 0.911 |
| number of inpatient stays; m (sd) | 7.54 | (10.11) | 8.36 | (11.18) | 6.63 | (8.85) | 0.484 |
| inpatient stay in the 12 months prior recruitment; n (%) | 14 | (21.2%) | 5 | (14.3%) | 9 | (29.0%) | 0.144 |
| schizophrenic disorders (F20-F29 according to ICD-10); n (%) | 40 | (57.1%) | 21 | (56.8%) | 19 | (57.6%) | 0.945 |
| affective disorders (F30-F39 according to ICD-10); n (%) | 26 | (37.1%) | 15 | (40.5%) | 11 | (33.3%) | 0.533 |
| neurotic, stress or somatoform disorders (F40-F48 according to ICD-10); n (%) | 16 | (22.9%) | 8 | (21.6%) | 8 | (24.2%) | 0.794 |
| disorders of adult personality and behaviour (F60-F69 according to ICD-10); n (%) | 10 | (14.3%) | 4 | (10.8%) | 6 | (18.2%) | 0.379 |
| more than one mental disorder; n (%) | 35 | (50.0%) | 17 | (45.9%) | 18 | (54.5%) | 0.473 |
| medication due to mental illness in the 18 months prior recruitment; n (%) | 64 | (91.4%) | 32 | (86.5%) | 32 | (97.0%) | 0.118 |
| psychosocial impairment (HoNOS)*; m (sd) | 10.16 | (6.17) | 10.54 | (7.27) | 9.73 | (4.71) | 0.588 |

n = absolute frequency; % = relative frequency; m = mean; sd = standard deviation of the mean / Pearson Chi^2^ test for categorical variables and t-test for continuous variables; *outcome variables

**Table S3: clinical characteristics related to physical health at baseline**

|  | **Total** | | **CAU** | | **HELPS** | | **P** |
| --- | --- | --- | --- | --- | --- | --- | --- |
| one or more physical diseases; n (%) | 48 | (68.6%) | 22 | (59.5%) | 26 | (78.8%) | 0.082 |
| Body Mass Index*; m (sd) | 32.63 | (8.27) | 31.11 | (7.15) | 34.29 | (9.17) | 0.112 |
| overweight (BMI ≥ 25 kg/m^2^)*; n (%) | 57 | (82.6%) | 28 | (77.8%) | 29 | (87.9%) | 0.269 |
| obesity (BMI ≥ 30 kg/m^2^)*; n (%) | 39 | (56.5%) | 18 | (50.0%) | 21 | (63.6%) | 0.254 |
| waist circumference associated with increased risk of metabolic complications*; n (%) | 62 | (92.5%) | 32 | (91.4%) | 30 | (93.8%) | 0.718 |
| Waist-to-Hip Ratio*; m (sd) | 0.97 | (0.09) | 0.97 | (0.09) | 0.98 | (0.10) | 0.732 |
| alcohol consumption more than once a month*; n (%) | 11 | (15.7%) | 6 | (16.2%) | 5 | (15.2%) | 0.903 |
| smoking*; n (%) | 39 | (55.7%) | 18 | (48.6%) | 21 | (63.6%) | 0.208 |
| sufficient oral hygiene*; n (%) | 21 | (30.0%) | 9 | (24.3%) | 12 | (36.4%) | 0.273 |
| physical well-being (FEW16)*; m (sd) | 2.79 | (1.22) | 2.82 | (1.20) | 2.77 | (1.26) | 0.874 |
| physical activity (h/d) (diary)*; m (sd) | 0.89 | (1.65) | 0.87 | (1.53) | 0.91 | (1.81) | 0.937 |
| Sports Quality Index (frequency questionnaire)*; m (sd) | 12.45 | (5.92) | 12.86 | (6.65) | 11.97 | (5.00) | 0.542 |
| Dietary Quality Index (frequency questionnaire)*; m (sd) | 30.07 | (6.01) | 31.25 | (6.20) | 28.79 | (5.59) | 0.089 |
| Healthy Dietary Indicator (diary)*; m (sd) | 3.29 | (1.20) | 3.21 | (1.26) | 3.37 | (1.15) | 0.615 |
| Health-related locus of control*; m (sd) | 37.77 | (8.37) | 39.89 | (7.87) | 35.39 | (8.39) | **0.024** |

n = absolute frequency; % = relative frequency; m = mean; sd = standard deviation of the mean / Pearson Chi^2^ test for categorical variables and t-test for continuous variables; significant effects (p <0.05) are printed in bold; increased health risk due to waist circumference defined as waist circumference ≥ 94 cm for men and ≥ 80 cm for women; adequate oral hygiene defined as brushing teeth at least twice a day for ≥ 2 min *outcome variables

**Table S4: Results of mixed-effects regression models for secondary outcomes**

| **Body Mass Index** | B | se | P | 95% - CI | | |
| --- | --- | --- | --- | --- | --- | --- |
| intercept | 29.8795 | 2.4665 | **<.0001** | 24.9564 | - | 34.8026 |
| group | 2.4677 | 2.2693 | 0.2794 | -2.0325 | - | 6.9679 |
| time | 0.1446 | 0.2036 | 0.4804 | -0.2631 | - | 0.5523 |
| time * group | -0.0959 | 0.2841 | 0.7364 | -0.6594 | - | 0.4675 |
| PS | 3.5044 | 5.2068 | 0.5024 | -6.8208 | - | 13.8297 |
| **Waist-to-Hip Ratio** | B | se | P | 95% - CI | | |
| intercept | 0.9796 | 0.0254 | **<.0001** | 0.9290 | - | 1.0302 |
| group | 0.0251 | 0.0266 | 0.3467 | -0.0276 | - | 0.0779 |
| time | 0.0064 | 0.0051 | 0.2130 | -0.0038 | - | 0.0166 |
| time * group | -0.0075 | 0.0071 | 0.2941 | -0.0216 | - | 0.0066 |
| PS | -0.0424 | 0.0475 | 0.3742 | -0.1368 | - | 0.0519 |
| **Dietary Quality Index (frequency questionnaire)** | B | se | P | 95% - CI | | |
| intercept | 31.0657 | 1.6545 | **<.0001** | 27.7623 | - | 34.3691 |
| group | -2.5559 | 1.6732 | 0.1297 | -5.8740 | - | 0.7622 |
| time | -0.0273 | 0.3266 | 0.9337 | -0.6810 | - | 0.6264 |
| time * group | 0.2057 | 0.4610 | 0.6564 | -0.7085 | - | 1.1199 |
| PS | 0.4802 | 3.1147 | 0.8778 | -5.6963 | - | 6.6568 |
| **Healthy Dietary Indicator (diary)** | B | se | P | 95% - CI | | |
| intercept | 2.9551 | 0.3876 | **<.0001** | 2.1790 | - | 3.7312 |
| group | 0.4288 | 0.4147 | 0.3054 | -0.4010 | - | 1.2586 |
| time | 0.2259 | 0.1167 | 0.0596 | -0.0096 | - | 0.4613 |
| time * group | -0.1481 | 0.1592 | 0.3560 | -0.4667 | - | 0.1705 |
| PS | -0.0422 | 0.6890 | 0.9514 | -1.4208 | - | 1.3365 |
| **Sports Quality Index (frequency questionnaire)** | B | se | P | 95% - CI | | |
| intercept | 12.7718 | 1.6123 | **<.0001** | 9.5536 | - | 15.9900 |
| group | -0.6853 | 1.5964 | 0.6687 | -3.8542 | - | 2.4836 |
| time | -0.3142 | 0.2904 | 0.2838 | -0.8957 | - | 0.2673 |
| time * group | 0.2040 | 0.4141 | 0.6235 | -0.6181 | - | 1.0260 |
| PS | -0.0561 | 3.2095 | 0.9861 | -6.4268 | - | 6.3147 |
| **Physical activity (h/d) (diary)** | B | se | p | 95% - CI | | |
| intercept | 0.9882 | 0.468 | **0.0393** | 0.0503 | - | 1.9261 |
| group | 0.0995 | 0.5254 | 0.8504 | -0.9511 | - | 1.1501 |
| time | -0.1137 | 0.1025 | 0.2737 | -0.3208 | - | 0.0934 |
| time * group | 0.0216 | 0.1499 | 0.8861 | -0.2781 | - | 0.3212 |
| PS | -0.1727 | 0.8163 | 0.8331 | -1.8050 | - | 1.4595 |

group = mean difference between control and intervention group at baseline

time = linear change from t0 to t3 in control group

time * group = difference in linear change between control and intervention group

PS = estimated coefficient for propensity-score adjustment
